# Feasibility of in vivo small animal imaging using a clinical total-body PET/CT system

**DOI:** 10.1186/s40658-025-00782-z

**Published:** 2025-07-23

**Authors:** Julia G. Mannheim, Wenhong Lan, Maurizio Conti, Franziska Siedler, Marcel A. Krueger, Kristina Herfert, Christian la Fougère, Fabian P. Schmidt

**Affiliations:** 1 Werner Siemens Imaging Center, Department of Preclinical Imaging and Radiopharmacy, Eberhard-Karls University Tuebingen, Roentgenweg 13, 72076 Tuebingen, Germany; 2 Cluster of Excellence iFIT (EXC 2180) “Image Guided and Functionally Instructed Tumor Therapies”, University of Tuebingen, Tuebingen, Germany; 3 Department of Nuclear Medicine and Clinical Molecular Imaging, University Hospital Tuebingen, Tuebingen, Germany; 4 Siemens Medical Solutions USA, Inc., Molecular Imaging, Knoxville, TN USA

**Keywords:** Total-body PET, Biograph Vision Quadra, Small animal imaging, In vivo quantification, Long axial FOV

## Abstract

**Background:**

Clinical PET scanners have long been explored for preclinical imaging, but limited spatial resolution and sensitivity have restricted their use for preclinical studies. The recent availability of total-body (TB) PET/CT scanners with extended axial fields of view (FOVs) has largely overcome sensitivity limitations, enabling potential new opportunities for small-animal imaging. This study evaluated the feasibility and performance of the Biograph Vision Quadra TB-PET/CT for rodent imaging compared to the dedicated small-animal PET scanner Inveon DPET.

**Material and methods:**

Recovery coefficients (RC), image noise, and optimum image reconstruction parameters were assessed using the preclinical NEMA NU 4–2008 image quality phantom and a sub-cohort of three anesthetized mice as a proof-of-concept demonstrating the feasibility of the setup. In vivo quantification accuracy was evaluated by scanning nine frozen mice simultaneously in three different arrangements with the Quadra compared with individual scans at the Inveon. To ensure comparability, all mice were snap-frozen after 1 h uptake of [^1^⁸F]FDG, scanned sequentially and individually at the Inveon (90 min p.i.), and subsequently scanned at the Quadra with decay-corrected acquisition times. SUV_mean_ and SUV_max_ values were determined for liver, muscle and brain regions on both systems. To evaluate potential position-dependent effects within the extended axial FOV, a single frozen mouse was scanned at multiple positions.

**Results:**

Phantom rods ≥ 2 mm could be resolved with the Quadra, showing a comparable RC for larger structures, e.g. for the 5 mm rod of 1.17 compared to 1.09 (Inveon) when using point-spread-function modeling, whilst having lower noise of 5.1%SD vs 9.0%SD. No substantial position-dependent effects were detected in the phantom or single-mouse scan across the axial FOV. SUV_mean_ values were consistent between both scanner across all investigated organs, with liver and muscle uptake remaining comparable for frame durations down to 5 s. SUV_max_ values exhibited greater variability, with significant differences observed in muscle and brain regions.

**Conclusion:**

Despite the lower spatial resolution of the clinical TB-PET/CT scanner (~ 3–4 mm) compared to the dedicated preclinical scanner (~ 1.5 mm), robust SUV_mean_ quantification was achievable. Together with successful in vivo imaging of anesthetized mice, these findings support the feasibility of using clinical TB-PET/CT for preclinical research, acknowledging spatial resolution as a limiting factor.

## Background

Small animal positron emission tomography (PET) imaging has evolved into a state-of-the-art method for investigating metabolic pathways and molecular targets for multiple diseases, such as oncology, neurology and inflammation [1]. This approach allows for longitudinal in vivo studies that are crucial for advancing our understanding of these diseases.

Following the development of the first dedicated small-animal PET scanners [2–4], these systems became more widely available and commercialized by the late 2000s/early 2010 s and since then, preclinical PET research has experienced a tremendous increase. However, preclinical PET research facilities are costly [5], and the maximum number of animals that can be scanned might be limited depending on tracer availability, as well as field of view (FOV) size constraints (typically a maximum of four mice or two rats per scan [6–9]), along with limitations in the noise equivalent count rate (NECR).

Therefore, clinical PET scanners with standard axial fields of view (FOVs) of 15–22 cm were evaluated for their potential use in preclinical PET research [5, 10–14]. Early studies have demonstrated that while these clinical systems provided decent image quality and potentially enabled qualitative analyses, e.g., information on the presence or absence of 2-deoxy-2-[^18^F]fluoro-D-glucose ([^18^F]FDG) uptake in tumors, they required a certain lesion size to enable accurate quantification [12, 13]. Tatsumi et al. reported that a quantitative analysis was possible for tumors larger than 2 cm if no recovery correction was available and for tumors larger than 1 cm if recovery correction was available [13], whereas Aide et al. reported that analyses of mouse models with tumors larger than 7 mm are feasible if reconstruction protocols with point spread function correction are available [12]. However, more advanced studies, such as those involving voxel-wise analysis in neurology research, are not feasible because of the limited spatial resolution and sensitivity of clinical PET systems [12, 13]. Furthermore, the limited spatial resolution (~ 3–4 mm compared to ~ 1 mm for preclinical scanner) results in an underestimation of the measured activity due to the increased partial volume effect (PVE) [11, 15]. The PVE is especially problematic in small animals such as mice and rats, where organ regions of interest are considerably smaller than those in humans. For example, the mouse brain has a volume of approximately 0.5 cm^3^, whereas the human brain has a volume of approximately 1200 cm^3^ [16, 17]. Hence, high spatial resolution and high sensitivity are required to accurately detect and quantify uptake patterns in small animals. Recently, total-body (TB) PET scanners in combination with high-resolution computed tomography (CT) have become increasingly available [18–20]. This new generation of scanners exhibits a high increase in sensitivity due to the extended axial FOV of up to 2 m, as well as an improved spatial resolution compared with the PET systems from the early 2000 s, for which more advanced reconstruction and correction protocols are available. This development raises the possibility of utilizing these TB-PET/CT scanners to perform high-quality preclinical research studies. The advantage of utilizing such clinical TB-PET/CT scanners for preclinical studies would be the possibility of considerably increasing the number of animals per scan (potentially more than 10 animals per scan, provided anesthesia and warming requirements can be sufficiently addressed to ensure stable animal temperatures and anesthesia supply), potentially requiring only a single PET acquisition to scan an entire animal cohort and thereby reducing intrascan variability and tracer costs.

Furthermore, though TB-PET/CT scanners are not as widespread as standard clinical PET scanners with a standard axial FOV, the number of installations has steadily increased in recent years. Considering that not every clinical center has access to a dedicated and costly preclinical imaging facility, those who have already transitioned to or are planning to purchase a TB-PET/CT scanner could benefit from the opportunities such a scanner might provide for preclinical research.

In this study, the performance of a clinical TB-PET/CT scanner Biograph Vision Quadra (Siemens Healthineers, Knoxville, TN, USA) for preclinical imaging was assessed for different image reconstructions and compared with a dedicated preclinical PET scanner Inveon DPET (Siemens Healthineers, Knoxville, TN, USA) using the preclinical NEMA NU 4-2008 image quality (IQ) phantom. The impact of reconstruction parameters was furthermore determined for a sub-cohort of 3 anesthetized mice that were scanned on the clinical TB-PET/CT scanner, which also demonstrated the feasibility of the experimental setup.

Potential influences of the animal positions within the FOV of the clinical TB-PET/CT scanner were evaluated to determine position-dependent differences in uptake due to changes in sensitivity and spatial resolution along the extended axial FOV [21]. In vivo quantification accuracy and comparability with the dedicated preclinical PET scanner was determined for a cohort of nine frozen mice scanned simultaneously on the clinical TB-PET/CT scanner.

## Methods

### Preclinical and clinical PET scanners

Animal and phantom scans were performed using a preclinical dedicated PET scanner (Inveon DPET, Siemens Healthineers, Knoxville, TN, USA) and a clinical TB-PET/CT scanner (Biograph Vision Quadra, Siemens Healthineers, Knoxville, TN, USA).

The preclinical Inveon DPET scanner, referred to as “Inveon” throughout this work, spans an axial FOV of 12.7 cm and a transaxial FOV of 10 cm; the reported spatial resolution is 1.63 mm full width at half maximum (FWHM, at a 5 mm radial offset, axial center field of view (cFOV)), and sensitivity of 6.72% (67.2cps/kBq), 4.0% and 2.8% for a point source, mouse and rat phantom, respectively [22]). Attenuation correction was performed with an external Co-57 source. The clinical TB-PET/CT system, referred to as “Quadra” throughout this work, spans an axial FOV of 106 cm and a transaxial FOV of 78 cm; the reported radial spatial resolution is 3.35 mm (at 1/2 of the axial FOV (53.0 cm) and a radial offset of 1 cm), the total sensitivity is 176 cps/kBq, and the time-of-flight resolution is 228 ps [18]. Attenuation correction is performed with the enclosed CT system. A further description of the utilized scanners can be found in the literature [18, 23].

### Animal studies

All animal experiments were approved by the local governmental agency (Regierungspräsidium Tübingen, Germany) and were performed according to local regulations. In total, 13 healthy female C3H mice (35.31 g ± 5.93 g) were utilized. The animals were maintained on a 12:12-h light‒12-h dark cycle, supplied with unlimited autoclaved food and water and were not fasted on the day of scanning. The mice were anesthetized with 1.5% isoflurane vaporized in oxygen gas at a rate of 1.0 L/min using a dedicated vaporizer (Vetland, Louisville, KY, USA). The animals were intravenously injected with 11.57 MBq ± 1.82 MBq of [^18^F]FDG via a tail vein catheter. Detailed information on body weight, injected activities and activities at the start of the respective acquisition can be found in Table 1.

**Table 1:** Detailed information on animal weights, injected activities and activities at the start of the respective acquisitions for the three anesthetized mice (a), the single frozen mouse (b) and the nine frozen mice scanned simultaneously (c)

	Weight [g]	Injected activity [MBq]	Activity at scan [MBq]	Frame duration [s]
a				
mouse 1	30.2	12.37	8.74	600
mouse 2	30.5	12.42	8.77	
mouse 3	24.2	12.47	8.74	

	Weight [g]	Injected activity [MBq]	Activity at scan [MBq]	Frame duration [s]
b				
Inveon	31.13	13.04	7.38	139
cFOV5			3.47	299
cFOV4			3.19	326
cFOV3			2.95	352
cFOV2			2.75	379
cFOV1			2.50	417
TransPos1			2.28	504
TransPos2			2.08	549
TransPos3			1.91	600

Mouse ID	Weight [g]	Injected activity [MBq]	Activity at Inveon scan [MBq]	Frame duration Inveon scans [s]	Activity at Quadra line scan [MBq]	Frame duration Quadra line [s]	Activity at Quadra grid scan [MBq]	Frame duration Quadra grid [s]	Activity at Quadra stacked scan [MBq]	Frame duration Quadra stacked [s]
c										
1	43.86	13.86	7.85	338	1.38	2119	0.83	3831	0.40	10,800
2	38.40	13.44	7.62		1.34		0.80		0.39	
3	42.56	13.76	7.79		1.37		0.82		0.40	
4	36.07	10.81	6.12	410	1.30		0.78		0.38	
5	31.08	10.31	5.84		1.24		0.75		0.36	
6	33.56	10.73	6.08		1.29		0.78		0.38	
7	39.57	8.87	5.02	497	1.29		0.77		0.38	
8	42.81	9.20	5.21		1.34		0.80		0.39	
9	35.04	9.07	5.14		1.32		0.79		0.39	

### Anesthetized animals

**Fig. 1:**
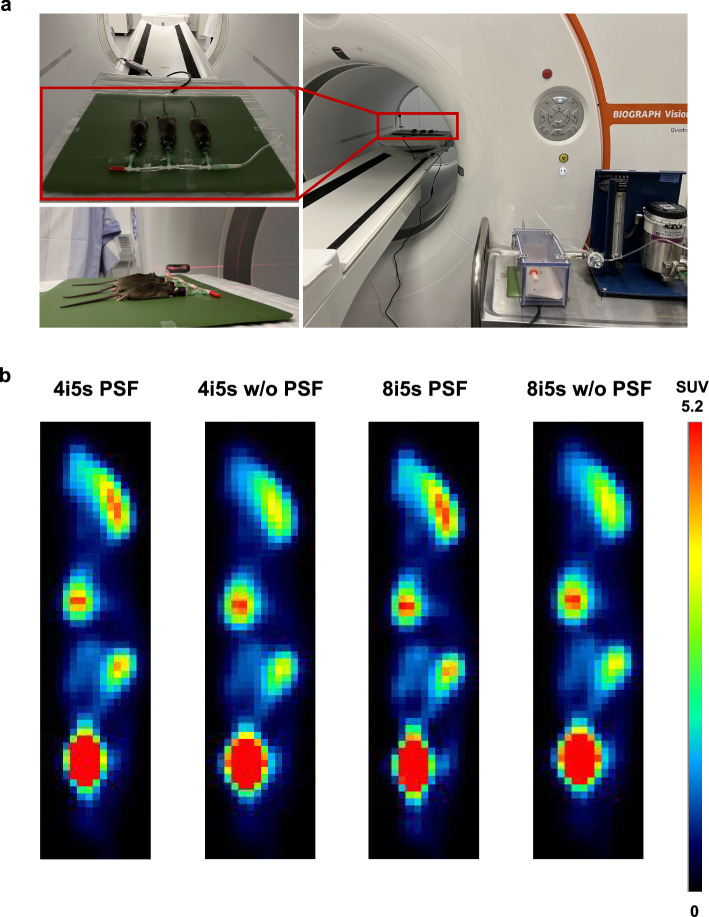
Experimental setup of the three anesthetized mice scanned on the Quadra scanner (**a**) and sagittal views of an anesthetized mouse scan performed on the Quadra scanner (**b**). Data were reconstructed with 4 and 8 iterations, respectively, with 5 subsets, and with and without PSF modeling

A sub-cohort of three anesthetized mice were maintained under anesthesia in a heated anesthesia box for 1 h uptake time, subsequently transferred to the Quadra scanner and placed on heating pads to maintain body temperature during the Quadra scan (see Fig. 1).

### Frozen animals

After the injection, similar to the three anesthetized mice, the mice were maintained under anesthesia in a heated anesthesia box for 1 h uptake time. Afterward, the mice were sacrificed in a CO_2_ box and snap-frozen in the same position using cooled isopropyl alcohol in combination with dry ice. The temperature of the alcohol was continuously monitored (temperature sensor PCE-T317; PCE Deutschland GmbH, Meschede, Germany) during the snap-freezing process to ensure stable temperatures of ~ −55 °C. The frozen mice were then individually placed in 3D-printed boxes (120 × 60 × 60 mm^3^) with dry ice surrounding the animals to ensure the same position during both the preclinical and clinical PET scans.

### Experimental setup, data acquisition and image reconstruction

**Fig. 2:**
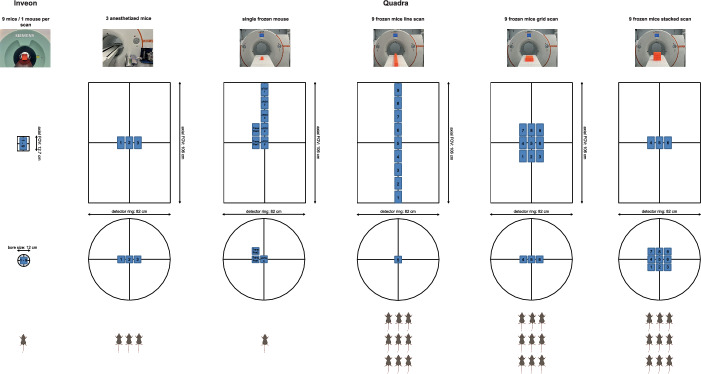
Schematic overview of the acquisitions performed for the analyses of the three anesthetized mice, the single frozen mouse and the nine frozen mice using the Inveon and Quadra scanners. Created with BioRender.com

- NEMA NU 4–2008 image quality (IQ) phantom: Following the NEMA requirements, the phantom was filled with 3.7 MBq of ^18^F and measured for 1200 s positioned at the cFOV of the Quadra. To evaluate position-dependent impact on detectability and recovery coefficients for the five rods (diameter 1,2,3,4 and 5 mm), additional scans prolonged to account for the decay were performed at a transaxial offset of 8 cm (axial center) and an axial offset of 48 cm (transaxial center). For comparison the IQ phantom filled with 3.7 MBq of ^18^F was scanned for 1200 s at the cFOV of the Inveon scanner.
- Three anesthetized mice: A 600 s emission scan was performed for the three anesthetized mice on the Quadra scanner.
- Single frozen mouse: To evaluate potential position-dependent uptake within the extended axial FOV of the Quadra scanner, a single frozen mouse was scanned at multiple positions within the FOV (see Fig. 2). The list mode data were acquired for 600 s for each position. Based on the last acquisition (TransPos3 position, center of the box at 120 mm axial and 60 mm transaxial offset from the cFOV), the frame durations of the previous acquisitions were shortened in the histogramming step to account for the physical decay, with the TransPos3 scan serving as a reference (see Table 1b for the respective acquisition times for all the scans). For comparison, a static emission scan of 139 s followed by an 803 s transmission scan using the Co-57 source was performed on the Inveon scanner.
- Nine frozen mice: All nine frozen mice underwent individual PET emission scans on the Inveon scanner followed by a transmission scan to correct for attenuation. Therefore, a subset of three mice was simultaneously scanned on three Inveon scanners in parallel. The injected activity was lowered for the second and third sets of mice to obtain equal activities for each mouse in the scans using the Quadra scanner. The acquisition time was accordingly increased from 338 to 420 s and 497 s for the scans of sets 1, 2 and 3, respectively (Table 1c). The mice were subsequently transferred to the Quadra scanner. List mode acquisitions were performed with multiple arrangements of the nine frozen mice scanned simultaneously (see Fig. 2 for the experimental setup) within the extended axial FOV as follows:

- Line scanall nine frozen mice were arranged in a line to cover the entire 106 cm axial FOV of the TB-PET/CT scanner; animal 5 was positioned in the transaxial and axial cFOV.

- Grid scan - three rows with three mice each were placed one behind the other, row 2 was positioned in the axial cFOV, and animal 5 was positioned in the transaxial and axial cFOV.

- Stacked scan - three rows with three mice each were placed on top of each other, row 2 was positioned in the axial cFOV, and animal 5 was positioned in the transaxial and axial cFOV.

The listmode data were acquired for 3 h for the stacked scan; the frame durations of the previous scans were calculated to account for the physical decay, with the stacked scan serving as a reference (see Table 1c for the respective acquisition times for all evaluated scans).

**Fig. 3:**
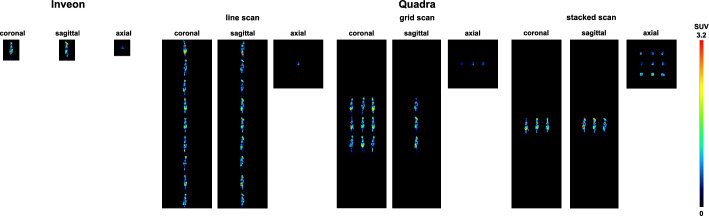
Qualitative comparison of the Inveon individual scans compared with the nine frozen mice simultaneously scanned with the Quadra scanner in different arrangements (line scan, grid scan, and stacked scan)

Figure 3 displays axial, coronal and sagittal views of the line, grid and stacked scans, showing all nine frozen mice scanned simultaneously within the extended axial FOV of the Quadra scanner compared with individual acquisitions on the Inveon scanner.

All acquisitions with the Quadra scanner were performed using its maximum axial acceptance angle of 52°, and all possible lines of responses were recorded. CT scans were performed for each position to correct for attenuation (120 kVp tube potential, automatic tube current modulation with 210 mAs ref.).

In addition, the line scan of the nine frozen mice was rebinned into shorter frame durations (600 s, 60 s, 30 s, 10 s and 5 s) to determine the potential impact on the quantification accuracy due to different count statistics.

Phantom data were reconstructed with an ordinary Poisson ordered subset expectation maximization (OP-OSEM) algorithm with 5 subsets and up to 10 iterations, with and without point spread function (PSF) modeling and with time-of-flight (TOF) information. The image matrix size was 440 × 440 × 645 with a voxel size of 1.65 × 1.65 × 1.65 mm^3^.

Anesthetized mice were reconstructed with the reconstruction protocol OP-OSEM with 4 and 8 iterations, respectively, with 5 subsets with and without PSF modeling and with TOF information. For the frozen mice, the reconstruction OP-OSEM with 4 iterations and 5 subsets (4i5s), PSF modeling and TOF information was chosen based on the results of the phantom data.

Preclinical data were reconstructed using the maximum a posteriori algorithm in combination with the 3D ordered subset expectation maximization algorithm (MAP/OSEM3D; 18 MAP iterations, beta smoothing value 0.053, 2 OSEM3D iterations and 18 subsets) with a matrix size of 256 × 256 × 159, resulting in a reconstructed voxel size of 0.388 × 0.388 × 0.796 mm^3^. The preclinical images were analyzed without a filter and with a Gaussian filter with a 1.63 mm kernel size (calculated based on the formula of Conti and Rothfuss [24]) to match the voxel size of the clinical PET system with the intention of improving comparability between the two systems.

### Data analysis

#### NEMA NU 4–2008 IQ phantom

A qualitative comparison of phantom images acquired with the Inveon and Quadra was performed to evaluate rod detectability and image noise across the three positions in the FOV, varying reconstruction parameters (4i5s and 8i5s), and the impact of PSF modeling. Quantitative accuracy was assessed by calculating recovery coefficients (RCs) and percent standard deviation (%SD) as a measure of image noise, following the NEMA protocol for all rods and across all iterations. Calculations were conducted using an in-house-developed MATLAB script (R2024a, The MathWorks, Natick, USA).

#### Animals

For the frozen mice, the Quadra PET scans were coregistered to the Inveon scans. For the single frozen mouse scans, a single volume of interest (VOI) was placed in the liver using the software tool PMOD (version 4.203, PMOD Technologies LLC, Zurich, Switzerland). For the nine frozen mice, VOIs were placed in the liver, muscle (musculus biceps femoris) and whole brain. Liver and muscle VOIs were outlined on the Inveon images and transferred to the co-registered Quadra images. For the whole brain region, the Quadra images were coregistered to a T2-weighted magnetic resonance (MR) template (provided by PMOD). A VOI for the whole brain region was created using the isocontouring tool in PMOD and used for the analysis of all datasets. For the three anesthetized mice, liver and muscle VOIs were contoured on the Quadra images. The liver VOIs were placed in the same part of the liver to enable comparison and exclude potential impacts of larger vessels.

Table 2 lists the respective VOI sizes of the analyzed organs, including the number of fractional voxels for each VOI. The SUV_mean_ and SUV_max_ were calculated accordingly for each region. Statistical analysis was performed using Prism 9.2.0 software (GraphPad, La Jolla, CA, USA). One-way ANOVA was performed with Tukey’s correction for multiple testing and Bonferroni’s correction for multiple organ testing on the same datasets; the statistical significance level was set to an adjusted p-value of ≤ 0.05.

**Table 2:** Detailed information on the VOI sizes of the analyzed organs, including the number of fractional voxels for each VOI

	Volume [cm^3^]	Number of fractional voxels	
		Inveon	Quadra
Liver	0.068	569.6	15.21
Muscle	0.016	129.2	3.5
Whole brain	0.549	6465.61	172.65

In addition, the PET data from the Quadra line scan were reframed to 2119 s, 600 s, 60 s, 30 s, 10 s and 5 s to evaluate the impacts of shorter acquisition times on the SUV_mean_ of the liver and muscle regions. A cropped sagittal PET image (1 × 46 × 73 voxels) of the mouse located at the center position of the Quadra line scan was used to assess the differences at the voxel level for the different frame durations. Difference images [%] were determined as the relative difference between each image with a shorter frame duration and the reference image with a 2119 s frame duration. The outline of the mouse was delineated to obtain a mask, which was applied to the difference images to eliminate the spurious impact of noisy voxels outside of the mouse. Subsequently, variations were quantitatively assessed by calculating the means and standard deviations of the voxel values of the difference images.

## Results

### NEMA NU 4–2008 IQ phantom

**Fig. 4:**
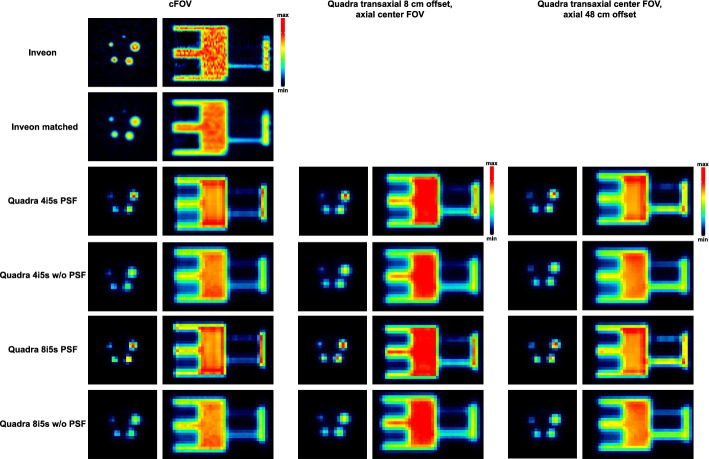
Qualitative comparison of the preclinical NEMA NU 4-2008 IQ phantom scanned on the Inveon and the Quadra system. Quadra scans were performed at the cFOV, with a transaxial offset of 8 cm at the axial center FOV, and with an axial offset of 48 cm at the transaxial cFOV. Quadra data were reconstructed with 4 and 8 iterations, respectively, with 5 subsets, and with and without PSF modeling

The transverse view of the IQ phantom (Fig. 4) demonstrates that the smallest 1 mm rod was clearly discernible in the Inveon images, whereas it could not be resolved in the Quadra acquisitions. The remaining rods (2–5 mm) were identifiable for the Quadra across all positions, including the transaxial and axial offset. Incorporation of PSF modeling in the image reconstruction improved the depiction of the rods in the Quadra images, resulting in enhanced edge definition and visible activity gradients, rendering them more comparable to the Inveon images.

**Fig. 5:**
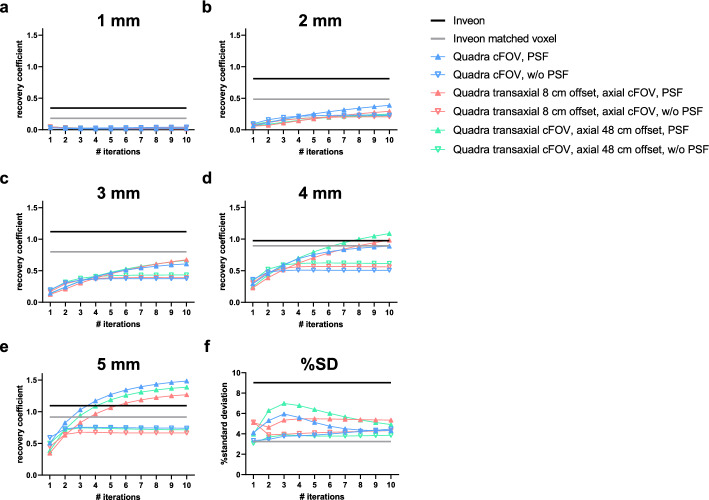
Quantitative analysis of the recovery coefficients for different rod sizes and the percent standard deviation (%SD) for the preclinical NEMA NU 4-2008 IQ phantom scanned on the Inveon and the Quadra system. Quadra scans were performed at the cFOV, with a transaxial offset of 8 cm at the axial center FOV, and with an axial offset of 48 cm at the transaxial cFOV. Each point represents one iteration, and the number of iterations increases from left to right

Despite being visually detectable, RCs for the 2 mm and 3 mm rods were substantially lower in the Quadra compared to the Inveon (Fig. 5). For example, the RC for the 2 mm rod at the cFOV (4i5s, PSF) was 0.21 for the Quadra, compared to 0.81 for the Inveon. Filtering the Inveon images to match the Quadra voxel size reduced the RC to 0.49, improving comparability between scanners.

Towards larger rods, RC values increased and showed a better comparability between both scanners, e.g., RC for the 5 mm rod was 1.09 (Inveon) compared to 1.17, 0.97 and 1.09 for the Quadra with 4i5s PSF and at cFOV, transverse and axial offset positions, respectively. Reconstructions without PSF yielded lower RCs, e.g., 0.51 for the 5 mm rod at the cFOV. For these larger structures, increasing the number of iterations resulted in activity overestimation, with, e.g., an RC of 1.43 for the 5 mm rod (cFOV 8i5s PSF).

While PSF-based reconstruction improved quantitative accuracy, it was associated with increased image noise, e.g., at the cFOV with 4i5s was 5.1%SD (PSF) compared to 3.8%SD (without PSF). Still, due to inherently lower spatial resolution of the Quadra, noise levels remained below those observed in the Inveon images (9.0%SD). This was visually evident in Fig. 4, where the Inveon image exhibited more pronounced noise, which could be mitigated to 3.3%SD by applying the filter.

### Animals

**Fig. 6:**
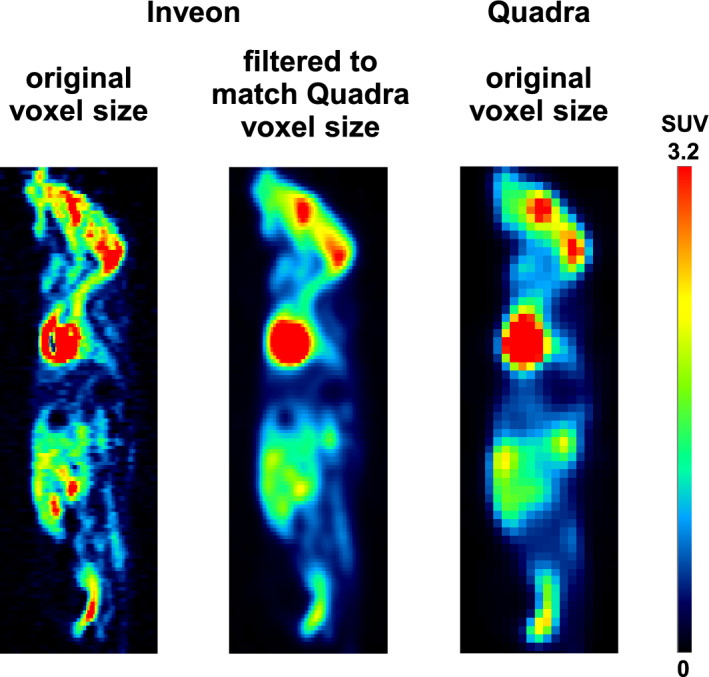
Sagittal views of a representative mouse scan with the original voxel size of the Inveon scanner (0.388 × 0.388 × 0.796 mm^3^), Inveon image data filtered (1.63 mm kernel) to match the Quadra voxel size, and Quadra voxel size (1.65 × 1.65 × 1.65 mm.^3^)

Figure 6 depicts a comparison of representative images of the same mouse scanned on the Inveon scanner and the Quadra scanner. The preclinical scan was reconstructed with the original voxel size and filtered to match the clinical PET voxel size. As expected, the preclinical scan with the original voxel size revealed a more distinct uptake pattern due to the improved spatial resolution compared with the clinical scanner, whereas the preclinical scan filtered to match the clinical voxel size revealed similar uptake patterns to the clinical scan.

### Three anesthetized mice

**Fig. 7:**
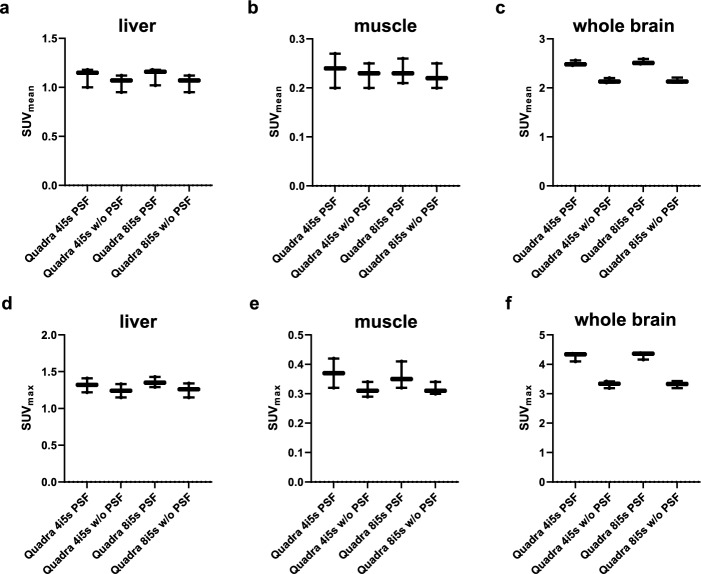
Quantitative analysis of SUV_mean_ and SUV_max_ for the liver (**a**,** d**), muscle (**b**,** e**), and whole brain (**c**,** f**) of the three anesthetized mice scanned using the Quadra scanner. Data were reconstructed with 4 and 8 iterations, respectively, with 5 subsets, and with and without PSF modeling

Figure 1a displays the experimental setup of a sub-cohort of three anesthetized mice scanned on the Quadra scanner. Representative images of one animal reconstructed with 4 and 8 iterations and with and without applying PSF modeling, respectively, are shown in Fig. 1b. Qualitative analysis demonstrates that the number of iterations only has a negligible effect on the images, whereas the PSF modeling has a distinct impact. Quantitative analysis of SUV_mean_ (Fig. 7a-c) and SUV_max_ (Fig. 7d-f) confirms this. Similar SUV_mean_ and SUV_max_ values were determined for 4 and 8 iterations with and without PSF modeling, respectively. Based on the phantom and sub-cohort animal data, the reconstruction protocol of 4 iterations with PSF modeling was chosen as best trade-off in RCs and noise for the subsequent evaluation of comparability between the scanners.

### Single frozen mouse

**Fig. 8:**
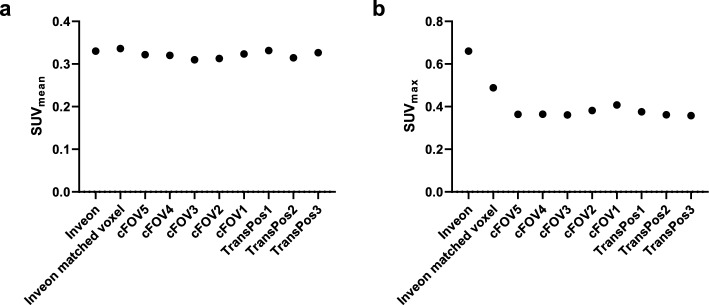
Quantitative analysis of liver SUV_mean_ (**a**) and SUV_max_ (**b**) of a single frozen mouse scanned at multiple positions within the Quadra FOV. Uptake was compared to the Inveon original voxel size, and the Inveon voxel size filtered to match the Quadra voxel size

Analyses of the SUV_mean_ and SUV_max_ for the liver in a single frozen mouse scanned at multiple positions within the Quadra FOV compared with the Inveon scan (both original and matched voxel sizes of the Quadra) are presented in Fig. 8a and b, respectively. Comparable liver SUV_mean_ values were determined, with 0.32 ± 0.01 (mean ± standard deviation) for all Quadra scans and 0.33 and 0.34 for the Inveon scans with original and matched voxel sizes, respectively. This finding demonstrates the comparability of the SUV_mean_ between all Quadra and Inveon scans, as well as the negligible impact of the position in the FOV of the Quadra scanner on the quantification accuracy. Comparable SUV_max_ values were obtained for the Quadra scans at multiple positions (mean for all Quadra scans: 0.38 ± 0.02), and increased SUV_max_ values were determined for the Inveon scans with original (0.66) and matched (0.49) voxel sizes.

### Nine frozen mice

**Fig. 9:**
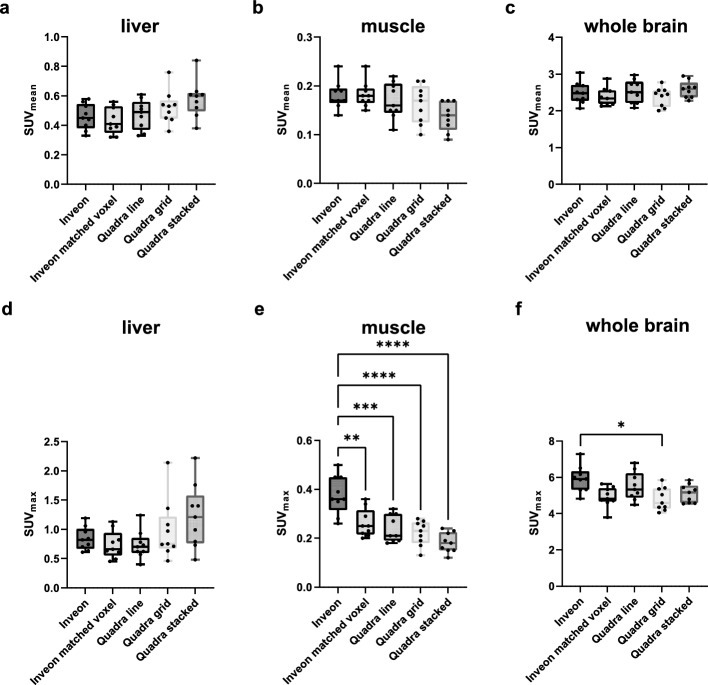
Quantitative analysis of SUV_mean_ and SUV_max_ for the liver (**a**,** d**), muscle (**b**,** e**), and whole brain (**c**,** f**) of the nine frozen mice scanned simultaneously using the Quadra scanner (line, grid and stacked scan). Uptake was compared to Inveon scans performed individually for each mouse (Inveon original voxel size and Inveon voxel size filtered to match the Quadra voxel size). One-way ANOVA was performed with Tukey’s correction for multiple comparisons and with Bonferroni’s correction for multiple organ comparisons in the same datasets

Figure 9 depicts the analyses of the SUV_mean_ and SUV_max_ for the liver (a, d), muscle (b, e), and whole brain (c, f), respectively, of the nine frozen mice scanned simultaneously with the Quadra system (line, grid and stacked scans) compared with the individual scans performed on the preclinical Inveon scanner (original and voxel size matched to the Quadra). Comparable SUV_mean_ between the Inveon and Quadra scans were obtained for all investigated regions. Furthermore, the liver SUV_max_ values were comparable among all the scans. For the muscle, significant differences in the SUV_max_ between the Inveon original voxel size images (0.38 ± 0.08) and all other scans (Inveon matched voxel: p = 0.007; Quadra line: p = 0.0007; Quadra grid: p < 0.0001; Quadra stacked: p < 0.0001) were detected. However, matched voxel size images (0.26 ± 0.06) revealed comparable SUV_max_ to those of all Quadra scans (line: 0.24 ± 0.06, grid: 0.22 ± 0.05, stacked: 0.18 ± 0.04). For the whole brain region, comparable SUV_max_ between the Inveon and Quadra scans were observed except for the Quadra grid scan (Inveon: 5.01 ± 0.73, Quadra grid: 4.80 ± 0.64; p = 0.0408).

**Fig. 10:**
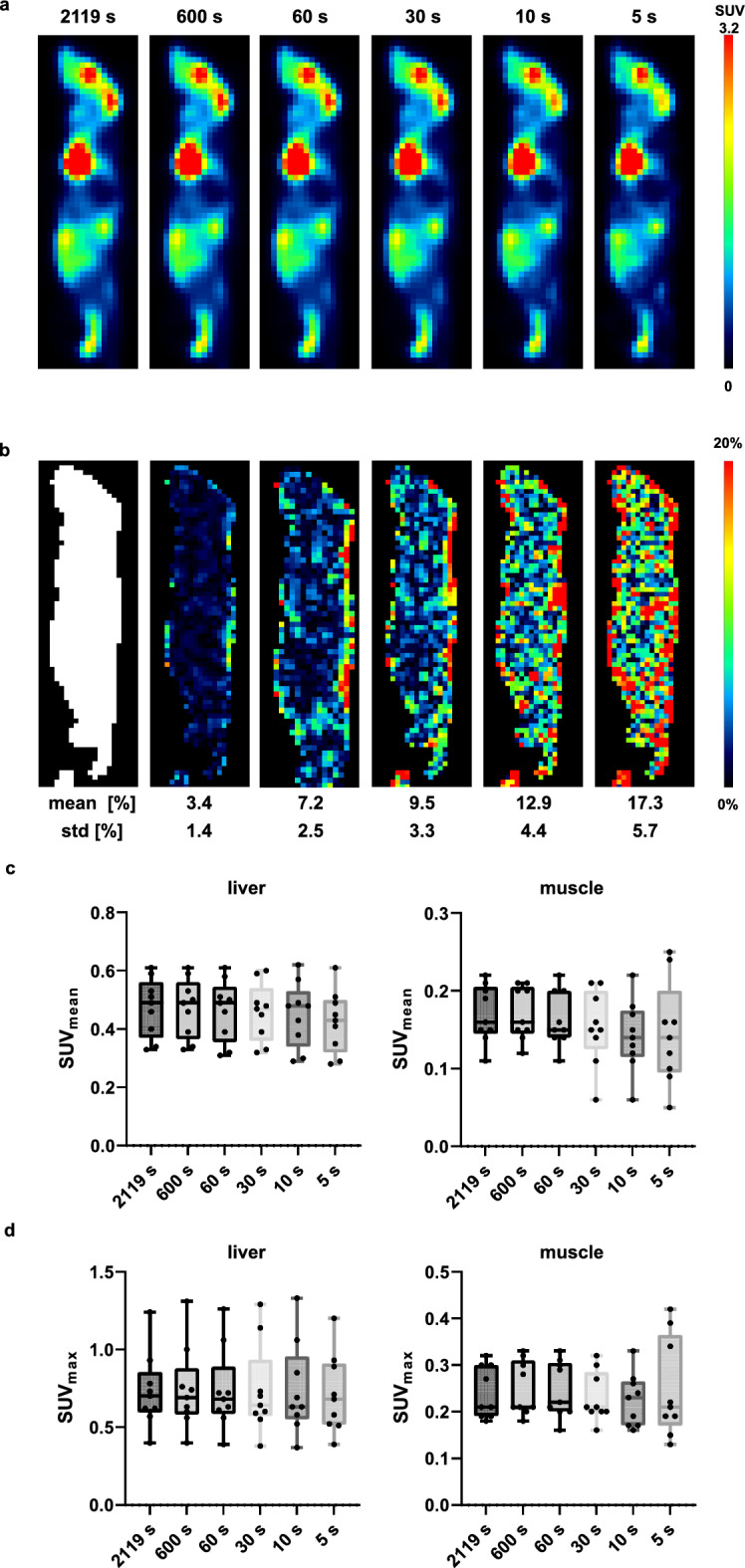
Qualitative comparison of different frame durations for a representative mouse scanned on the Quadra (**a**), mask and relative difference images with reference to the 2119 s image frame (**b**). The means ± standard deviations [%] are presented for the percent difference between the respective frames and the reference frame. Quantitative analysis of the SUV_mean_ (**c**) and SUV_max_ (**d**) of the liver and muscle of the nine frozen mice scanned simultaneously with the Quadra system (line scan) using the different frame durations. One-way ANOVA was performed with Tukey’s correction for multiple comparisons and with Bonferroni’s correction for multiple organ comparisons using the same datasets

A qualitative comparison of the representative mouse images captured at different frame durations (Fig. 10a) revealed comparable uptake patterns for frame durations down to 30 s. For the 10 s and 5 s frames, minor differences were visible, e.g., within the brain region. The relative difference images (Fig. 10b) demonstrated larger differences at the voxel level toward shorter acquisition times, e.g., 3.4 ± 1.4% 9.5 ± 3.3% and 17.3 ± 5.7% for 600 s, 30 s and 5 s, respectively. Furthermore, for all investigated frame durations, comparable SUV_mean_ in the liver and muscle were obtained (Fig. 10c); however, the standard deviation increased with decreasing frame durations due to the decrease in count statistics, e.g., liver 16.3 ± 3.2 (600 s) and 14.6 ± 3.6 (5 s), and muscle 6.0 ± 1.4 (600 s) and 5.1 ± 1.8 (5 s).

## Discussion

This study evaluated the feasibility of using a clinical TB-PET/CT scanner for preclinical small animal imaging. Image quality and comparability in quantification were assessed with the NEMA NU 4–2008 IQ phantom and in a sub-cohort of three anesthetized mice, followed by simultaneous scans of nine frozen mice with the Biograph Vision Quadra scanner that were compared with sequential scans with the Inveon preclinical PET scanner.

The phantom study confirmed the expected limitations of the clinical TB-PET/CT scanner in resolving submillimeter structures, with the 1 mm rod remaining undetectable due to the inferior spatial resolution of the Quadra (3.35 mm FWHM at ½ of the axial FOV and 1 cm radial offset [18]) compared to the Inveon (1.63 FWHM at the axial center FOV and 5 mm radial offset [23]). Nonetheless, rods ≥ 2 mm could be identified, and for the 4 mm and 5 mm rods, good agreement in RCs was observed between both systems. This agreement persisted across different positions within the field of view, including transaxial and axial offsets, where potential degradation due to parallax error or reduced sensitivity showed minor impact (e.g., RC 4 mm rod 0.69 (cFOV) and 0.62 (48 cm axial offset)). Of note, PSF modeling substantially improved quantitative accuracy partially compensating for the system’s lower spatial resolution. These findings highlight that while the Quadra is not suited for resolving very small structures, still quantification for larger structures is enabled. For additional information on position dependent spatial resolution and contrast recovery, we refer to our previous studies in which the Quadra was characterized at 18 positions within the FOV, extending the NEMA NU 2–2018 protocol using a Na-22 point source [21], and contrast recovery evaluations using small hot-sphere phantoms with warm background under various reconstruction settings [25].

As a proof-of-concept to demonstrate feasibility of the experimental setup and to evaluate the impact of reconstruction parameters, a sub-cohort of three anesthetized mice was scanned on the Quadra scanner. Higher SUV_mean_ and SUV_max_ values were determined when PSF modeling was applied, which is in line with the phantom data. No impact of the number of iterations was determined on SUV_mean_ and SUV_max_ values.

A potential position-dependent uptake for a realistic in vivo scenario was evaluated with a single frozen mouse scanned at multiple positions within the Quadra FOV (Fig. 2). Analogue to the phantom studies, the qualitative analysis (Fig. 6) revealed a higher resolution of smaller animal structures for the Inveon scan, as expected, due to the different spatial resolutions of the two scanners.

The quantitative analysis revealed comparable liver SUV_mean_ and SUV_max_ at all investigated positions within the Quadra FOV (Fig. 8), indicating that varying the spatial resolution and sensitivity along the FOV had no impact on the SUV measurements [18, 21]. The SUV_mean_ data from the Quadra scans were also consistent with the Inveon SUV_mean_ data. However, the SUV_max_ was considerably higher for the Inveon scans (0.66) than for the Quadra (0.38 ± 0.02) scans. This difference can be attributed to the lower image noise (5.1%SD) of the Quadra (cFOV, PSF, 4 iterations), supported by the blurring associated with the lower spatial resolution, compared with 9.0%SD noise for the Inveon.

In order to achieve the best possible comparability of semiquantitative uptake values between different PET scanners or even between the same scanners within multicenter studies, harmonization is required. This process requires the definition of standardized scanner-specific image reconstruction and scan protocols, including phantom scans, scanner calibration and quality control, e.g., for clinical PET scanners, according to the European Association of Nuclear Medicine (EANM) FDG PET/CT guidelines for tumor imaging [26]. However, although various attempts and strategies have been proposed for preclinical PET scanners [27, 28], preclinical harmonization remains a challenge [29]. We investigated a simplified approach by matching the small voxel size of the Inveon scanner to the larger voxel size of the Quadra scanner by applying a Gaussian filter. This method helped to obtain a better visual comparison of the two scans (Fig. 6) and a better agreement for the liver SUV_max_, with values of 0.49 (Inveon matched voxel size) and 0.38 ± 0.02 (Quadra).

Interestingly, we did not observe any position-dependent differences in the SUV parameters along the Quadra FOV. In previous work, we reported that due to the parallax error, the average spatial resolution (e.g., 4.1 mm FWHM (axial center) vs. 3.6 mm FWHM (40 cm axial offset)) [21] and contrast recovery (e.g., 56% (axial center) vs. 62% (40 cm axial offset)) [25] vary along the FOV. However, in this study, although the liver is a small structure, the SUV parameters were not affected, presumably as the respective VOIs covered only a fractional central part of the liver.

An entire animal cohort of nine frozen mice was scanned simultaneously with the Quadra instrument, and the SUV_mean_ and SUV_max_ measurements of different target regions were compared with sequential scans performed on the Inveon scanner. Comparable SUV_mean_ to those of the Inveon scanner were determined for all investigated organs (Fig. 9a-c). This result is particularly remarkable for the investigated muscle region, as the respective VOIs in the Quadra datasets contained only ~ 3.5 fractional voxels compared with 129.2 fractional voxels for the Inveon dataset. Notably, this low number of voxels determined the same quantitative uptake, although the Quadra voxels were resampled to the Inveon voxel sizes (VOIs were delineated on the Inveon datasets and transferred to the Quadra scans). Furthermore, the relatively large group strength of the 9 animals utilized in this study improved the comparability between the Quadra and Inveon scanners.

The SUV_max_ in the liver were comparable between the clinical and preclinical scanners (Fig. 9d). Even with the limited number of voxels, e.g., 15 voxels for the liver VOI, relatively homogeneous [^18^F]FDG uptake [30] is assumed to result in a comparable SUV_max_ between the two scanners. For the muscle SUV_max_, significant differences were observed between the Inveon and Quadra scans. However, by matching the Inveon voxel size, a comparable SUV_max_ of 0.26 ± 0.06 (Inveon matched voxel size) to the values of all Quadra scans could be obtained (Fig. 9e).

For the larger whole brain region, comparable SUV_max_ were also determined between the Inveon scanner and the Quadra scanner (except for the Quadra grid scan, Fig. 9f). However, inhomogeneous uptake patterns in small brain regions, especially with PET tracers targeting central nervous system receptors [17], can lead to significant spill-in and spill-out effects due to high binding in specific brain regions, such as the striatum, and low binding in surrounding areas. This pattern, combined with a low number of voxels, can result in larger deviations in the SUV_mean_ and SUV_max_ for smaller brain regions.

In addition, functional PET studies of mice or rats, where differences in small brain regions are determined or a voxel-wise analysis via parametric mapping is applied, will be impaired by the lower spatial resolution of the Quadra scanner. Nonetheless, investigations of anesthesia effects or treatment responses at the whole-organ level would be feasible for mice and rats.

The quantitative analysis of the different frame durations for the Quadra (Fig. 10) revealed that for frame durations down to 5 s, the SUV_mean_ and SUV_max_ was still comparable to the value of the reference frame of 2119 s, although the number of trues was highly reduced (for all 9 animals: 3.72 × 10^6^ (5 s) and 1.40 × 10^9^ (2119 s)). Furthermore, although the mean percentage deviation for the 5 s frame duration compared with the reference scan was 17.3%, the SUV_mean_ and SUV_max_ measurements in the liver and muscle regions were still comparable. However, the muscle region exhibited a larger standard deviation, which can be attributed to the smaller VOI size with fewer voxels included and hence a greater contribution of noise due to the limited count statistics. Notably, high quantification and image quality are still present even with short 5 s frames, suggesting a potential application for dynamic imaging. These parameters could be further improved by performing future scans at 60 min p.i. (instead of > 4.5 h, as in this study). The shortest frame duration for the Inveon was 139 s for the single frozen mouse experiment. When comparing SUV_mean_ and SUV_max_ values with a 600 s frame duration (typical static frame duration in our institute), the SUV_mean_ changed by only 1.8%, whereas the SUV_max_ revealed a change of 35.3% (data not shown). The SUV_max_ is more affected by technical variations and image noise then SUV_mean_, and is based on a single voxel value, potentially not reflecting the entire biology of the underlying structure [31]. The SUV_mean_ is characterized with an overall greater robustness and comparability in our study.

This work can be extended to rat imaging, where a further improvement in quantification is expected due to the larger organ structures compared with those of mice.

Finally, due to the large dimensions of the FOV of the Quadra scanner, an unrestrained, freely moving rat could be imaged in its home environment (cage), minimizing stress levels and allowing the analysis of unaffected brain responses [32], provided that an advanced motion correction method is used [33].

One constraint of our study is that, while PSF modeling was applied during reconstruction of the Quadra data—partially compensating for PVE—no such correction was applied to the Inveon data. Although this may have affected comparability, no PVE correction option was available within the standard Inveon reconstruction workflow. Furthermore, a comparison with ground truth data, e.g., gamma counting, was not available for absolute quantification and comparison of the activity concentrations, as the animals were snap-frozen to avoid motion artifacts during scanning and to allow motion-free comparison and coregistration of the Inveon and Quadra datasets. Notably, the evaluation of the single frozen mouse and nine frozen mice in this study is based on an ex vivo comparison and hence is not impacted by potential motion artifacts (cardiac and respiratory motion) that could impact the comparability of both Quadra and Inveon scans. In contrast to this, our proof-of-concept study of three anesthetized animals (see Figs. 1 and 7) is impacted by cardiac and respiratory motion accordingly and demonstrated the feasibility of the experimental setup.

Dedicated preclinical imaging systems are state-of-the-art tools for performing high-resolution and high-sensitivity preclinical research. Although the preclinical PET scanner used in the present study does admittedly not belong to the latest generation of available preclinical PET scanners, it nevertheless is widely in use and provides robust and reliable results, which is of utmost interest to ensure longitudinal comparability and reproducibility.

A major advantage of using a clinical TB-PET scanners for preclinical studies is the ability to scan entire cohorts of animals simultaneously within a single acquisition, e.g., for drug screening. This process, of course, requires an advanced anesthesia setup to provide each animal with a constant, reproducible and individual anesthesia supply, as well as advanced temperature monitoring to ensure stable animal temperatures throughout the scan.

Scanning an entire animal cohort in a single PET scanner eliminates the intrascan variability that typically occurs when scanning animal cohorts with individual sequential PET scans (e.g. due to statistical fluctuations, animals’ circadian rhythm and tracer production specificity), thereby improving the reliability of animal comparisons within a cohort. Imaging sites with a clinical TB-PET scanner that do not have access to a dedicated preclinical imaging facility may have new opportunities for preclinical research by exploiting the high sensitivity of the clinical TB-PET scanner.

## Conclusions

Although the spatial resolution is considerably different between preclinical and clinical PET scanners (~ 1.5 mm vs. 3–4 mm), performing comparable studies of SUV_mean_ in mice with a clinical total-body PET/CT system at the whole-organ level is feasible due to the significant increase in sensitivity. Our study demonstrated that clinical total-body PET/CT scanners can be used for preclinical in vivo imaging, with the unique advantage of being able to scan multiple animals simultaneously.

## Data Availability

The datasets used and/or analyzed during the current study are available from the corresponding author upon reasonable request.
